# Sepsis in Aging Populations: A Review of Risk Factors, Diagnosis, and Management

**DOI:** 10.7759/cureus.74973

**Published:** 2024-12-02

**Authors:** Abdulaziz H Alhamyani, Musharraf S Alamri, Nawwaf W Aljuaid, Abdulrhman H Aloubthani, Shafi Alzahrani, Ali A Alghamdi, Abdullah S Lajdam, Hamza Alamoudi, Abdulrahman A Alamoudi, Adham M Albulushi, Saad Nasser AlQarni

**Affiliations:** 1 Family Medicine, Armed Forces Hospitals, Taif, SAU; 2 Emergency Medicine, King Abdulaziz University, Jeddah, SAU; 3 Cardiology, King Fahad General Hospital, Al-Baha, SAU; 4 Internal Medicine, King Abdulaziz University, Jeddah, SAU; 5 Internal Medicine, Al-Baha University, Al-Baha, SAU

**Keywords:** aging populations, elderly care, geriatric medicine, immunosenescence, inflammaging, septic shock

## Abstract

Sepsis remains a significant global health concern, particularly among aging populations. This comprehensive review examines the complex interplay between aging and sepsis, focusing on risk factors, diagnostic challenges, and management strategies specific to older adults. The study explores the physiological changes associated with aging that contribute to increased sepsis susceptibility, including immunosenescence and chronic inflammation. It delves into the unique pathophysiology of sepsis in the elderly, highlighting the role of comorbidities and age-related immune dysfunction. The review emphasizes the importance of early recognition and tailored interventions for sepsis in older patients, addressing the often atypical presentation of symptoms in this population. By synthesizing current research and clinical guidelines, this study aims to enhance understanding of sepsis in aging populations and improve outcomes through targeted prevention, prompt diagnosis, and effective management strategies. The findings underscore the need for age-specific approaches in sepsis care and highlight areas for future research to address the growing burden of sepsis in an aging global population.

## Introduction and background

Sepsis is a life-threatening condition that happens when the body’s reaction to an infection triggers widespread inflammation, which can cause severe damage to tissues and organs [1]. In recent years, sepsis has emerged as a global public health threat, especially in people over the age of 70 years [2]. Older individuals are at increased risk of sepsis due to the presence of multiple comorbidities and other factors that compromise their immunity. Globally, sepsis proportions ranged from 30-32% among patients aged more than 60 years old [3]. Furthermore, sepsis is one of the top ten causes of death in the United States, affecting about 750,000 people each year, with half needing intensive care [4]. The word “sepsis” is derived from the Greek word “sepo,” which translates to ‘to rot’ [5]. In the past, sepsis was considered a severe disease that destroys the body, but in 1989, it was redefined as a syndrome encompassing many signs and symptoms caused by the body’s response to invading microorganisms. Bacterial, fungal, and parasitic agents can be the source of sepsis syndromes. Such agents produce inflammatory mediators as well as induce cellular dysfunction in the affected host [6,7]. Patients with sepsis may exhibit symptoms such as hypothermia or hyperthermia, tachycardia, tachypnea, and at least one end organ failure due to hypoperfusion [8]. This can lead to specific diseases affecting the nervous system, such as hypoxemia, increased plasma lactate, and oliguria [1]. 

In 1991, the American College of Chest Physicians (ACCP) and the Society of Critical Care Medicine (SCCM) redefined critical care medicine, leading to the emergence of a new term, systemic inflammatory response (SIRS) [9]. The diagnosis of SIRS requires meeting at least two of the following four clinical conditions: hyperthermia or hypothermia, tachycardia, and abnormal white blood cell count. In 2001, 29 international experts in the field of sepsis came together to define sepsis, critical illness, and septic shock [10]. They concluded that the previous definition from 1991 was still valuable for clinical and research purposes. However, they defined a specific category of sepsis; “severe sepsis” would be sepsis complicated by at least one organ failure, and “septic shock” would be severe sepsis with impaired blood flow leading to failure to respond to other causes of hypertension [10]. The participants in the consultation on sepsis definitions agreed that patients with sepsis should be classified by diagnosis and assessment according to four main factors: predisposing factors, infection, host response, and dysfunction [11].

As more people reach the age of 65 and older [12], the number of sepsis cases continues to rise. Therefore, it is important to understand how sepsis affects elderly people. Delays in recognizing sepsis can lead to severe complications, which makes timely intervention even more critical [13]. Despite growing awareness of sepsis in older adults, there are still significant gaps in research and practice. This review was conducted to summarize the key findings from the literature regarding sepsis in aging populations, including its risk factors, diagnosis, and management.

## Review

Pathophysiology

Sepsis in older adults follows a complex, often distinct path compared to younger individuals. Aging brings about numerous physiological changes, especially in immune response and organ function, that increase vulnerability to sepsis [2]. Sepsis is a life-threatening condition that is defined nowadays as organ dysfunction due to dysregulated host response to infection. The definition of organ dysfunction is a score of two or more by Sequential Organ Failure Assessment (SOFA) score [14]. More than half of sepsis cases are diagnosed in the elderly population aged 65 years or more [15]. The pathophysiology of sepsis in older adults involves immune system alterations, inflammation, and the resultant organ dysfunction. One of the cornerstones in the pathophysiology of sepsis among aging populations is immunosenescence, which refers to the aging of the immune system [16]. This phenomenon is assumed to be an essential factor contributing to what is called “inflammaging,” a term used to describe chronic low-grade systemic inflammatory response [17]. Immunosenescence affects both innate and adaptive immunity [18-20]. For the innate immune system, aging brings a reduction in the ability to process and present antigens. The adaptive immune system, on the other hand, shows a decrease in T-cell receptor diversity and struggles to form strong immune memories, which makes it harder to fend off previously encountered pathogens [17]. When senescent immune cells adopt a pro-inflammatory profile, known as the senescence-associated secretory phenotype (SASP), they release elevated levels of inflammatory cytokines, immune regulators, growth factors, and proteases. This activity can accelerate the progression of tumors and other age-related diseases [21]. 

Elderly patients face a higher risk of infections due to various factors like malnutrition, multiple medications, limited mobility, skin ulcers, and the use of catheters or other foreign devices. They are also more vulnerable because of conditions like hormonal deficiencies, difficulty emptying the bladder completely, and blood clot risks that come with age [14,22]. In older adults, the equilibrium between pro-inflammatory and anti-inflammatory responses can become imbalanced, often resulting in a harmful “cytokine storm” that negatively affects tissue and organ function. This mismanaged inflammation increases the severity of sepsis and complicates the clinical treatment of elderly patients who are affected [23]. The interaction between immune system dysfunction and inflammation in sepsis can lead to serious organ failure. Older individuals often have existing health issues, such as cardiovascular and chronic kidney diseases, which complicate their body’s response to sepsis. In cases of septic shock, vital organs receive reduced blood flow due to widespread blood vessel dilation and increased capillary permeability, resulting in tissue oxygen deprivation and ultimately organ failure. Common outcomes include acute respiratory distress syndrome (ARDS), acute kidney injury (AKI), and liver dysfunction, all of which significantly increase the mortality rates related to sepsis in this population [24,25].

Causes

Sepsis has increasingly become a significant public health concern, partly due to the growing global aging population [15]. Globally, the number of people 60 and older is predicted to rise from 841 million in 2013 to over two billion by 2050 [26]. Furthermore, sepsis occurrence rates have risen 20.4% more quickly in older persons over 65 than in younger adults under 65 [27]. There are multiple factors that contribute to the increased risk of infections in older persons. Age-related changes in the expression and function of toll-like receptors, as well as a decrease in cytokine production, all seem to contribute to the body’s immunological response to infection [28]. Moreover, malnutrition increases an older person’s vulnerability to infections and has a role in immunological dysregulation. An additional risk factor for infection in the elderly is dementia, poor motor function, and a history of falls and accidents [29]. 

Elderly people may also be more susceptible to infection overall if they have other concomitant diseases such as diabetes mellitus, chronic obstructive pulmonary disease (COPD), chronic renal disease, and cancer [30]. In older persons, genitourinary tract infections and respiratory tract infections are the most frequent infectious origins of sepsis [27]. Escherichia coli was the most often detected bacterium (50%) in urine cultures from individuals who had sepsis from a urinary source [31]. Streptococci, enterococci, and Staphylococcus aureus are common gram-positive organisms seen in older persons with bloodstream infections. Approximately 17% of older persons (60 years of age) with sepsis from pneumonia had a positive culture for methicillin-sensitive S. aureus, and 12.3% had a positive culture for methicillin-resistant S. aureus (MRSA) [31].

Risk factors

Sepsis and septic shock are potentially fatal infections that are particularly dangerous for elderly patients because of several risk factors that increase susceptibility to these conditions in older age groups, including age-related physiological changes, pre-existing medical conditions, and comorbidities. 

Age

Age plays a significant role in the risk of developing sepsis. Age brings about various physical changes, and one of the most impactful changes happens in the immune system. For instance, older adults may experience a reduced production of immune cells, such as T cells and B cells [32]. Nutrition is another critical aspect that impacts the risk of sepsis in older adults. Many elderly individuals do not consume adequate nutrients, which can impair immune function [33]. With age, skin becomes thinner and loses elasticity, which makes it more susceptible to injury and infection [34]. Skin is supposed to serve as a barrier to the entry of any possible pathogen load. Older adults may have cuts or sores that heal slowly, which can provide an opportunity for infections to take hold. More so, other age-related skin problems such as pressure ulcers are more common among the elderly, particularly those with poor self-care abilities like mobility [35]. These wounds can become infected and lead to a higher risk of sepsis. Moreover, a significant number of elderly patients who suffer from neuropathy experience a lot of reduction in tissue sensation [36,37]. This can make them ignore small wounds, thinking they are insignificant, resulting in the untreated wounds getting severely infected. Many older individuals may not present with classic symptoms, such as fever or elevated white blood cell counts [38]. 

Age-related immune modifications

One of the key aspects for the development of sepsis among older people is termed immunosenescence, a normal physiological process of aging that diminishes the immune response. As people grow older, their immune system becomes less robust [39]. This results in a compromised defensive system. In older adults, the body’s inflammatory response, which is essential for warding off infections, is frequently weaker, raising the risk of systemic infection [40,41]. The thymus, an organ critical for producing T cells, shrinks over time [42]. As a result, the output of new T cells declines. This reduction means that older adults have a smaller pool of immune cells capable of recognizing and responding to new infections. In addition, the function of T cells diminishes with age [39]. CD4+ T helper cells, essential for orchestrating the immune response, show decreased proliferation and production of key cytokines [43]. This decline leads to an impaired ability to activate B cells [44]. Consequently, the antibody response to vaccines and infections becomes less effective. Older adults may not produce adequate levels of antibodies in response to vaccinations like influenza and pneumonia [45,46]. The innate immune system acts as the body’s first line of defense against infections. With age, the innate immune system becomes increasingly unbalanced, which often results in chronic inflammation and various declines in immune cell function [47]. Immune cells start to produce fewer cytokines when faced with the right antigens, and their ability to present antigens diminishes as well. Other processes, like phagocytosis and chemotaxis, slow down, reactive oxygen species (ROS) levels shift, and there is a continuous breakdown of cells without proper cellular renewal [48]. While older adults may still have similar numbers of immune cells, even into very advanced ages, these cells experience functional losses tied closely to aging [49]. 

For instance, neutrophils, which play a critical role in engulfing and destroying pathogens, exhibit altered functions. Older adults may experience a delay in neutrophil migration to sites of infection, which reduces their ability to combat invading microbes [14]. This is primarily caused by dysregulated signaling pathways related to protein kinases that can hinder the coordination and execution of phagocytosis [50]. In addition, increased oxidative stress as well as the production of ROS in aged patient neutrophils may be detrimental to the phagocytic function [51]. Furthermore, macrophages, which are also a vital element of the innate immune system, also appear to be malfunctioning in the elderly [52]. Monocytes and macrophages constitute a major response of the immune system to foreign organisms. They are capable of initiating and maintaining innate immune responses, such as phagocytosis and secretion of cytokines [53]. Macrophages experience significant changes in function as individuals age, but their overall numbers remain stable. In older adults, macrophages tend to have a reduced initial response to infections and inflammatory triggers [54]. This decrease may be due to decreased levels of toll-like receptors (TLRs) and associated signaling pathways caused by the aging process. According to a study done by Van Duin et al., it was found that old age affects the TLR-1, TLR-2, and TLR-4 stimulation, which leads to a decrease in active cytokines such as TNF-α, IL-1β, and IL-6 [55]. In sepsis patients, IFN-γ and GM-CSF treatment have been indicated to improve the functions of monocytes and macrophages, leading to improved phagocytosis of the pathogens [56,57]. Natural killer (NK) cells are vital for targeting and destroying virus-infected and tumor cells [58]. Sepsis patients tend to have a substantial decrease in the circulating NK cell count. This reduction correlates with poor prognosis because of decreased survivability rates, possibly as a result of increased cell death [59]. Studies suggest that the inability of the NK cells to be functional even when circulation levels are normal is the result of septic-induced immunosuppression. This condition makes patients more vulnerable to secondary infections and the reactivation of dormant viruses [60]. In the case of the elderly, it has been noted that NK cells are less effective at killing infected or tumor cells. This is probably due to the decreased chance of perforin being discharged at the immune synapse in the elderly [61,62].

Chronic inflammation

Chronic low-grade inflammation, often termed “inflammaging,” is another hallmark of aging [37]. This condition is characterized by persistent activation of the immune system, which leads to increased levels of pro-inflammatory cytokines and other inflammatory markers. While inflammation is a natural response to infection, chronic inflammation can impair the immune response and contribute to tissue damage [63]. In older adults, the presence of chronic diseases, such as obesity, diabetes, and cardiovascular disease, can exacerbate this inflammatory state. Inflammaging happens when certain receptors in the body lose their efficiency [37]. These receptors are responsible for picking up various types of damage signals, whether from external threats, the body’s own cells, or in-between states. When they sense these signals, they activate the innate immune system [63]. However, as these receptors start to wear down, the immune response becomes less controlled, leading to chronic, low-level inflammation as the body attempts to clear out accumulated "garbage" [64]. The ongoing inflammation can result in a cycle where the immune system becomes less effective at responding to new infections while simultaneously causing damage to healthy tissues. This situation complicates the management of sepsis, as the body struggles to mount an adequate defense against infections while dealing with the consequences of inflammation [38].

Long-term illnesses

Chronic conditions, including diabetes, heart disease, chronic renal disease, and chronic obstructive pulmonary disease (COPD), affect a large number of the senior population. These coexisting disorders affect multiple organ systems and impede the body’s ability to fight against infections. For instance, COPD patients are more likely to get respiratory infections, and diabetes can hinder the immune system’s ability to fight off infections. Sepsis is facilitated by the accumulation of many comorbidities, which increases the likelihood of infections overwhelming the body [65-67]. 

Diabetes and chronic kidney disease

Diabetes is one of the most common chronic diseases among older adults. It not only affects blood sugar levels but also has far-reaching effects on the immune system [68]. People with diabetes are 1.5 to four times more likely to suffer from infections compared to non-diabetic individuals [69]. Diabetic patients may suffer from neuropathy, reducing their ability to feel injuries or infections, especially in their extremities. When these infections go unnoticed or untreated, they can escalate to sepsis quickly [70]. Chronic kidney disease affects the body’s ability to filter waste and maintain fluid and electrolyte balance. As kidney function declines, so does the body’s ability to regulate blood pressure and respond to infections effectively [71]. Individuals with CKD often experience a weakened immune response, which makes them more susceptible to infections that can lead to sepsis [72]. Moreover, the treatments for CKD, including dialysis, can increase exposure to pathogens, further elevating the risk of infection. A large cohort study reported that CKD was clearly associated with sepsis [73].

Chronic obstructive pulmonary disease (COPD)

COPD is another prevalent condition among older adults, with approximately 12.64% of individuals over the age of 40 years suffering from this condition globally [74]. This disease hampers the airflow and the gaseous exchange processes within the lungs, thereby causing long-term breathing problems. Further, due to their low lung function, patients suffering from COPD have a greater proclivity for acquiring pneumonia and other respiratory ailments [75]. Hospitalization is often caused by flare-ups of COPD, which can further cause the risk of infections to rise drastically. The inflammation of the lung region can also lead individuals to an extreme immune response, which can potentially progress to sepsis [76,77].

Hospitalization and infections associated with healthcare

With advancing age, people are swamped with myriad health issues that predispose them to developing sepsis. Particularly, elderly people are more prone to hospital-acquired infections (HAIs) as they are hospitalized frequently and undergo invasive procedures such as catheterization or mechanical ventilation [78]. Hospitals are necessary for treating underlying medical conditions, but they also expose patients to organisms that are resistant to drugs [79]. One of the most frequent consequences of these infections is sepsis, especially in older individuals with poor immunity. This risk is further increased by invasive procedures, extended hospital stays, and ICU admissions [80,81]. HAIs include a range of infections that patients may acquire while receiving treatment for other conditions. The most common types of HAIs include urinary tract infections (UTIs), pneumonia, surgical site infections (SSIs), and bloodstream infections [79]. UTIs often occur due to the use of catheters, which can introduce bacteria into the urinary system. Older adults are at increased risk because they may require catheterization for extended periods [78]. Ventilator-associated pneumonia is a serious risk for older adults who require mechanical ventilation. Additionally, those with compromised lung function are at greater risk for developing pneumonia during hospitalization. Each of these infections can trigger a cascade of complications, ultimately leading to sepsis [79]. 

Clinical presentation 

In contrast to younger patients, older adults might not have fever or other typical infection symptoms. Instead, individuals may exhibit very slight temperature increases or hypothermia [82]. Modifications in mental state, such as disorientation, restlessness, or agitation, can be evident and could be confused with delirium or dementia [83]. Elevated heart rate and breathing rates are prevalent. At first, blood pressure may be normal or even high, but as sepsis worsens, low blood pressure may appear [82]. Changes in oxygen saturation levels or an increase in the effort required to breathe are signs of respiratory distress. Poor perfusion might be indicated by skin symptoms such as mottling, pallor, or cyanosis [82]. Generalized malaise and weakness are often present and can be mistaken for normal aging processes or other chronic conditions. Sepsis can result from urinary tract infections, which are frequent in the elderly. Instead of the typical flank discomfort or fever, symptoms may include dysuria, increased urgency, or impaired mental status [83]. 

Common organisms

Older individuals are more likely to be infected with gram-negative bacteria, and among individuals over 65, Escherichia coli is the main cause of community-acquired bacteremia known to cause urinary tract and intra-abdominal infections [84]. Furthermore, carbapenem-resistant Klebsiella pneumoniae (CRKP), especially in elderly patients, results in higher morbidity and mortality [85]. Similarly, elderly individuals have more than double the mortality and mortality rate from S. aureus bacteremia. In older adults, methicillin-resistant S. aureus infections have a worse prognosis than methicillin-sensitive S. aureus infections [86]. Mortality among individuals with invasive pneumococcal illness and sepsis is elevated in older age and higher severity of chronic diseases [87]. Candida species is the leading cause of opportunistic infections globally, primarily affecting individuals aged 65 and above [88]. Anaerobic bacteremia still has a relatively high death rate, particularly among the elderly [89]. 

Diagnosis

Sepsis is quite difficult to diagnose in the elderly population. Age-related immune system disorders, together with co-morbidities, often result in infections that are not normal in patients [90]. Clinical symptoms are usually mild. This makes laboratory diagnostics and on-site testing important for early detection [38].

Laboratory diagnostics for sepsis

Blood Culture

Blood cultures are still the gold standard for identifying the causative pathogens in sepsis. However, obtaining reliable results from elderly patients is often complicated by the late onset of bacterial infection and lower pathogen loads [91]. Studies show that blood culture sensitivity in the elderly is decreased, often leading to delays in starting appropriate antimicrobial therapy [38].

Procalcitonin (PCT)

Procalcitonin (PCT) is recognized as a biomarker for bacterial infections, with high levels indicating a strong possibility of sepsis. In the elderly population, the use of PCT testing offers the clinician a means of distinguishing between a bacterial cause and a nonbacterial cause of systemic inflammatory response syndrome [92]. Studies have indicated that PCT is especially useful in elderly patients since it is not greatly influenced by immunosenescence, unlike other markers such as C-reactive protein [93].

C-reactive Protein (CRP)

CRP has been used for a long time as a predictor of inflammatory as well as infectious processes. The elderly population may have a lower output of CRP in response to antigen due to weakened immunity. This may in turn cause an underestimation of the severity of the infection [94]. Furthermore, patients with chronic infections who have elevated levels of CRP may have arthritis or other cardiovascular diseases. This renders it a non-specific marker of sepsis [95]. 

Lactate

Lactate levels are an important tissue indicator of hypoxia and prognosis in sepsis. The elevated lactate in elderly sepsis patients was also linked to poorer outcomes [96]. This increase in lactate may be influenced by other factors, common conditions such as heart failure or respiratory failure [38]. Nonetheless, lactate still remains one of the elements important in the diagnosis of sepsis in the elderly [83].

Point-of-care diagnostics (POCT)

Point-of-Care Lactate Monitoring

The POC lactate test is a rapid and reliable method for diagnosing sepsis. In the elderly group, when the disease is diagnosed at an early stage, the outcome can be greatly improved [97]. Studies show that POC lactate measurements correlate well with parental laboratory values. It is a practical solution in intensive care facilities [98].

Rapid Pathogen Identification

Molecular diagnostics, such as polymerase chain reaction (PCR)-based methods, allow the rapid identification of pathogens in sepsis. POC molecular testing can directly test blood samples for bacterial DNA. This reduces the time required to identify the pathogen compared to traditional culture [99].

Biomarker Panels

Recent advances in POCT include multi-biomarker panels that can simultaneously detect infection-related markers such as interleukins, PCT, and PCR. These multi-biomarker panels provide outstanding diagnostic results within a few minutes [99].

Using POC lactate testing, rapid molecular diagnostics, and biomarker panels is improving diagnostic accuracy. As these technologies evolve, they hold the potential to further enhance sepsis management in elderly patients [100].

Management of sepsis in the elderly

Treatment of adults with sepsis or septic shock should align with Surviving Sepsis Campaign (SSC) guidelines, yet certain considerations are essential [101]. In antibiotic therapy, empirical treatment must account for potential pathogens and resistance patterns. The risk of multidrug-resistant infections is significant in this population due to common health problems [102]. In addition, the elderly are at increased risk of fungal infections due to age, weakened immune systems, catheter use, long-term antibiotic use, and medical conditions such as corticosteroids and chemotherapy [103]. Appropriate fluid management is essential in fluid resuscitation and hemodynamic support, given concurrent and age-related changes in autoregulation [104]. Although guidelines recommend a target arterial pressure (MAP) of 65 mm Hg, adults with chronic hypertension may need a higher target MAP to prevent renal disease [105]. Dehydration is common in adults and often requires initiation of a 500 mL crystalloid bolus. However, resuscitation policy may be problematic for patients with heart disease or renal disease, such as 30 mL/kg crystalloid administered over three hours [105]. 

The fluid response should be assessed dynamically, as excessive fluid therapy may have adverse effects [106]. Self-assessment of perfusion markers, including mental pressure, diuresis, blood flow testing, pulse rate, blood pressure, capillary refill, and bedside echocardiography, is vital for managing relapse. Prompt initiation of resuscitation with diuretics is essential [105]. Noninvasive ventilation (NIV) reduces the risks associated with mechanical ventilation and reduces discomfort in critically ill patients [107]. Although guidelines generally recommend NIV for the treatment of severe COPD with hypercapnia and acute respiratory failure due to pneumonia, it is not a first-line treatment for hypoxic respiratory failure due to pneumonia because intubation should be performed after NIV fails. Completed is a condition that has significant clinical implications and increases the risk of death [108]. 

Early recognition

Early identification of sepsis is critical, especially in older adults who may present atypically or have altered physiological responses [82]. Clinical markers such as changes in mental status, hypothermia or fever, tachypnea, and hypotension must be closely monitored. Utilizing tools like the quick Sequential Organ Failure Assessment (qSOFA) can help in screening for sepsis in a timely manner, leading to early intervention [41].

Antimicrobial therapy

Early initiation of broad-spectrum antimicrobial therapy is essential, as delays in appropriate antibiotic administration are associated with increased mortality [102]. In the elderly, dosing adjustments may be required due to altered pharmacokinetics and renal function. De-escalation of antibiotics based on culture results is advised to reduce the risk of antimicrobial resistance and adverse drug reactions [15].

Supportive care

The initial idea behind using corticosteroids to treat sepsis was based on the belief that sepsis results from an uncontrolled inflammatory response, and corticosteroids’ anti-inflammatory properties would improve outcomes [109]. This led to numerous clinical trials targeting various inflammatory pathways and mediators, such as endotoxin, toll-like receptors, and cytokines like TNF-α and interleukins. However, none of these trials produced an effective, licensed drug for sepsis, likely due to both the ineffectiveness of some agents and issues with patient selection and trial design [110]. Caring for sepsis in geriatric patients is generally much more extensive and may include the use of vasopressors in order to help with perfusion, the use of assisted breathing devices, and renal replacement therapy if necessary [111]. There is a need to constantly watch out for acute complications like acute respiratory distress syndrome (ARDS), acute kidney injury (AKI), and delirium. In addition, some measures that enhance immunity, including nutritional support and good blood sugar control, also have potential benefits [110].

Challenges and barriers to effective management 

In the past ten years, there has been an increase in the prevalence of severe sepsis, which is a prevalent and significant cause of morbidity and mortality in the elderly population. An estimated 750,000 persons in the US are predicted to suffer severe sepsis annually, with around 60% of those patients being older than 65 [112,113]. Rangel-Frausto et al. characterized the epidemiology of patients with two, three, or four SIRS criteria, sepsis, severe sepsis, and septic shock in a second prospective cohort analysis. A total of 2527 (68%) of the 3708 patients admitted to the survey units fulfilled the SIRS requirements. Among patients with SIRS, 649 (26%) developed sepsis, 467 (18%) had severe sepsis, and 110 (4%) experienced septic shock [114]. The elderly may have a delayed or missing early inflammatory response to infection, which often results in sepsis symptoms and signs. However, subsequent presentations may be quite severe and develop quickly into septic shock [22,115]. Furthermore, many individuals may lack a distinct history because of age-related dementia. Thus, diagnosing sepsis in this population requires a lower threshold and a larger index of suspicion [22]. Apart from the atypical reaction to infection, older patients’ lack of cooperation makes it difficult to get sufficient diagnostic specimens since they are weak, exhausted, incapacitated, and cognitively impaired [115].

Husabø et al. discovered a correlation between the duration it took to administer antibiotics and the completion or delay of diagnostic investigations [116]. According to adjusted studies, patients who were not triaged within 15 minutes experienced an additional 54.7 minutes of delay before receiving antibiotics on average. They discovered a similar pattern of longer times between the administration of antibiotics in cases where patients were not seen by a doctor within the triage timeframes of 40.8 to 81.6 minutes, 86.2 minutes for failing to have blood lactate measured within an hour, and 39.3 minutes for inadequate observation [116]. The combined prediction of all four techniques, when included in a single regression analysis, was a 159-minute delay before the first antibiotic dosage [116].

## Conclusions

This review highlights the unique challenges of diagnosing and managing sepsis in elderly patients, characterized by atypical presentations, altered physiological responses, and the influence of comorbidities. Early recognition and intervention are critical, as delays significantly increase mortality rates. The study underscores the utility of diagnostic tools such as procalcitonin and point-of-care testing in improving early detection. To enhance outcomes, a multidisciplinary approach integrating rapid diagnostics, appropriate antimicrobial therapy, and comprehensive supportive care is essential. Future research should aim to develop age-specific diagnostic criteria and treatment protocols for elderly sepsis patients.
